# Lift Every Voice: Engaging Black Adolescents in Social Justice Service-Learning to Promote Mental Health and Educational Equity

**DOI:** 10.1007/s11121-023-01570-w

**Published:** 2023-09-15

**Authors:** Sonya Mathies Dinizulu, Gabriel M. Velez, Mirinda Morency, Kristen Jacobson, Kelsey Moore, Nichole Carter, Stacy L. Frazier

**Affiliations:** 1 Department of Psychiatry & Behavior Neuroscience, University of Chicago, Chicago, IL USA; 2 College of Education, Marquette University, Milwaukee, WI USA; 3 Institute for Child Development, University of Minnesota, Minneapolis, MN USA; 4 Community Strategies, Health Management Associates, Chicago, IL USA; 5 Bright Star Community Outreach, Chicago, IL USA; 6 Department of Psychology, Florida International University, Miami, FL USA

**Keywords:** Black Adolescents, Feasibility, Social Justice, Mental Health, Service-Learning

## Abstract

This study reports on the feasibility and acceptability of a social justice infused service-learning (S-L) program to promote Black adolescent mental health and educational equity. We convened a community advisory board to help adapt and pilot test, via open trial mixed method design, an evidence-based service-learning program for Black middle school adolescents (*n* = 21) attending summer camp at a faith-based setting. We describe a S-L curriculum, with a focus on the achievement gap, and training for church staff and assess staff and youth reports of feasibility, acceptability, and promise to (a) improve/engage psychological engagement targets, and (b) improve academic motivation, and social-emotional and behavioral outcomes. Mixed method findings revealed high feasibility and acceptability of the S-L intervention as indicated by consistent attendance and enthusiastic engagement by staff and youth, high satisfaction, high completion rates of planned sessions, and emergent qualitative themes from staff interviews and adolescent focus groups highlighting that service-learning (1) facilitated skills (e.g., goal-setting, social-emotional and behavioral regulation, and problem-solving), (2) shaped perspectives and inspired openness, and (3) created a space for all to feel valued and included to address the inequities of education that directly impacted them. There was preliminary evidence for efficacy in that youth report of emotional symptoms, peer problems, and staff report of general internalizing symptoms decreased following the intervention, while youth report of prosocial behaviors increased. Implications suggest that S-L programming demonstrates promise to promote mental health outcomes, raise social awareness, and inspire critical consciousness and lift the voices of Black youth by providing tools for working toward systemic changes to reduce inequities in both education and mental health.

**Supplementary Information:**

The online version contains supplementary material available at 10.1007/s11121-023-01570-w.

## Introduction

The historical trauma of systemic racial oppression has negatively affected the well-being and mental health of Black youth and their families. Adverse effects include depressive, anxiety, and trauma symptoms (Priest et al., 2013), greater psychological distress (Gaylord-Harden & Cunningham, 2009), poorer self-esteem, decreased academic achievement and engagement, greater engagement in externalizing behaviors, risky sexual behaviors, and substance use (Benner et al., 2018). Across time, peaceful nation-wide uprisings against oppression have involved significant participation by young people, often met with resistance from White supremacist or racist groups (Hope & Spencer, 2017; Watts et al., 2011). Youth who engage in peaceful protests, activism, civic engagement, and service projects seek to strengthen their local communities and better society at large (Ginwright et al., 2007; Morimoto & Friedland, 2013). Young people’s social justice advocacy also benefits their individual well-being (Diemer et al., 2016; Ginwright & Cammarota, 2002). Service-learning (S-L) is an increasingly popular mechanism for advocacy, and sustained mental health and academic benefits are well documented for civically engaged (mostly White) college students. Less is known about the opportunities and benefits of S-L for Black adolescents affected directly by systemic racial oppression (Hope & Spencer, 2017). This study examined the feasibility, acceptability, and benefits of a 10-week summer program focused on closing the achievement gap for Black middle schoolers in a Black church.

### Black Churches Support Black Youth

Black churches offer programming—including treatment and clinical care, supports, and information—to benefit physical, mental, and civic health (DeKraai et al., 2011). Most promote adult wellness, which indirectly benefits children (DeHaven et al., 2004; Reingold et al., 2007), or support young people’s education (religious instruction and academic support; Bielefeld & Cleveland, 2013). Increasing attention in faith-based communities is going toward programming for ethnic and racial minority youth in high poverty urban communities, especially during unsupervised times when youth are exposed to risk (e.g., gang-related violence, illegal substances, unsafe sexual behaviors). During the past two decades, Faith-Based Organizations (FBO) increasingly have provided after-school and summer programs responding to family and community needs for safe, supervised, and enriching out-of-school time programming (U.S. Department of Education, 2008).

Additionally, Black churches routinely encourage and provide space for discussing civic issues and actions, for example, advertising marches and protests or hosting speakers from social service and community agencies (e.g., Brewer et al., 2003; Chaves, 2004). Black churches have a long history of mobilizing for political and social action to address community needs (e.g., Brown & Brown, 2003), facilitating generalizable skills (e.g., writing, planning and organization, presenting), and enabling leaders to emerge within and beyond the faith setting (e.g., Cavendish, 2000).

Beyond the traditional legacy of advocacy and activism, the critical historical and cultural role Black churches play for developing young people often transcends adversity. Spirituality can be viewed as a coping mechanism for all people, but African Americans are more likely to endorse the use of spiritual coping behaviors (e.g., praying, attending church services), especially for dealing with racism, chronic poverty, violence, health issues, and trauma. During times of distress, youth of color relied on prayer and “faith talk” to deepen connection with and trust in a higher being to see them through (Dill, 2017). Gardner (2011) further argued that religious affiliation and spirituality may protect and promote positive development for urban youth. Indeed, given their long-standing role supporting Black youth via advocacy and civic engagement efforts, Black churches are well positioned to promote and sustain the benefits of service-learning programs and initiatives.

### Service-learning to Promote Social Justice and Mental Health

S-L can be designed to elevate social justice for youth, build civil society, and promote positive youth development. At its traditional core, S-L is a structured method of instruction, integrated into students’ academic curriculum, by which youth learn through active participation in service experiences that meet their community’s most urgent and critical needs. By its deliberate focus on social awareness, S-L emphasizes community problem-solving through critical thinking about the roots of social inequality, and encourages youth to examine and influence historical, social, political, and economic decisions that sustain, exacerbate, mitigate, or resolve social inequities (Ginwright & Cammarota, 2002). A social justice and social awareness approach encourages youth to disrupt inequitable systems that impact learning and participation.

S-L is most impactful when students assume ownership over both service and learning (Fredericks, 2003) through a youth-led experiential process (based on experiential learning theory; Kolb, 1984; Kolb & Kolb, 2011) that moves through cyclical stages of Investigation, Preparation, Action, Reflection, Demonstration (of learning) and Celebration, and Evaluation (IPARDC-E; NYLC, 2021). Students grow as they investigate community needs, plan and enact service activities, and reflect and share their learnings with the larger community. This cyclic process yields positive long-term benefits when students are engaged for a long period of time, e.g., an entire school year (Shek et al., 2021). Transformation continues as students, teachers, and communities grow and address emergent needs. Throughout, a social awareness approach encourages students to disrupt unjust systems and dismantle hierarchical power relations (e.g., provider/provided, powerful/powerless; Wade, 1997) by embracing a dynamic network of problem-posers and problem-solvers (Kinloch et al., 2015). Ultimately, youth become a valuable community resource through critical, active, formal, and informal civic participation (Watts & Flanagan, 2007). Projects with explicit social justice goals may teach students how to write letters to public officials, conduct surveys, engage peers, circulate petitions, outreach to community leaders, design a web page, or lobby legislators. S-L leverages civic engagement to advance understanding of and involvement in community organizing for a democratic, just, and equitable society that upholds the worth and potential of every individual.

Three of the eight S-L Standards for Quality Practice (NYLC, 2008)—reflection, academic instruction, and youth-adult partnerships (Y-AP)—relate to youth competencies and psychological engagement, and, in turn, promote social-emotional, behavioral, and academic outcomes. First, *structured reflection* (e.g., group discussions, journaling) bridges community service with learning objectives facilitating changes in personal and social outcomes compared to programs without reflection (e.g., Simons & Cleary, 2006). The quantity and quality of reflection predicted deeper understanding and better application of knowledge and increased complexity of problem and solution analysis (Ash et al., 2005). Second, longitudinal studies show that, compared to community service alone, infusing community service into college *academic instruction* enhances critical thinking and writing; improves complex understanding, problem analysis, and cognitive development (e.g., Yorio et al., 2012); raises college GPA; and improves personal and interpersonal development, leadership, and communication (e.g., Vogelgesang & Astin, 2000). Third, *youth-adult partnerships* that engage youth in decision-making and action promote self and organizational development (e.g., Kirshner et al., 2002). Longitudinal studies illustrate that teens with positive connections to teachers or other adults more often avoid risky health behaviors; conversely, disengaged or disenfranchised youth engage in more violence and risk-taking, and report more emotional distress (Sieving et al., 2017). Strong positive social ties promote youth competencies and psychological engagement, promoting successful academic, social, and behavioral outcomes for youth (Travis & Leech, 2014).

S-L inclusive of reflection, academic instruction, and youth-adult partnerships shows increasing promise to benefit youth academic (Fredricks et al., 2004; Furrer et al., 2006), social-emotional (e.g., Fredricks et al., 2004), and behavioral (e.g., Klem & Connell, 2004) outcomes, especially via youth competencies and psychological engagement. Aligned with theories of positive youth development and risk reduction (e.g., Guerra & Bradshaw, 2008), and with content inherent to health promotion (Flanagan, 2004), S-L facilitates problem-solving and conflict resolution, planning and organization, and leadership (e.g., Celio et al., 2011). Also, S-L fosters a sense of community belonging (Pak, 2018) and social responsibility (Scales et al., 2000), encouraging psychological (e.g., McGuire & Gamble, 2006) and civic (e.g., Lichtenstein et al., 2011) engagement.

For example, an open trial of a S-L curriculum delivered to high school students (*n* = 792; 27% African American) in an urban under resourced community revealed baseline to post-test declines in dropouts and fighting and increases in GPA and postsecondary education (Kinsley et al., 1999). A more rigorous trial of S-L in an urban, under resourced middle school where students (*n* = 118; 52% African American) reported stronger connections with teachers, school attachment, improved attitudes (toward non-violence) and behavior (fewer absences, fights) compared to peers in the randomly assigned comparison group (Sieving & Widome, 2008). Finally, a study of Youth Empowerment Solutions for Peaceful Communities (YES) including project implementation and reflection (not the full S-L curriculum) showed that YES middle school youth (*n* = 40; 97% African American) were less likely than controls to become victims of violence, use more conflict resolution and avoidance skills, and demonstrate leadership skills (Zimmerman et al., 2018).

### Current Study

**Fig. 1:**
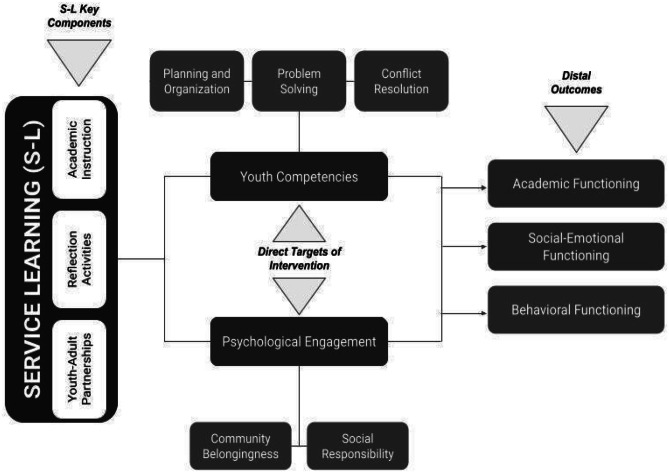
Conceptual model

The present study harnesses the unique potential of a Black church to integrate S-L into their youth programming, with a focus on the achievement gap. In this paper, we describe a S-L curriculum and training for church staff (toward a *sustainable model* rather than contracting out for service providers). Informed by step 1 of the Clinic/Community Intervention Development model (CID; Burns & Hoagwood, 2002), we also assess staff and youth reports of feasibility, acceptability, and promise to (a) improve/engage psychological engagement targets and (b) improve academic motivation, and social-emotional and behavioral outcomes, for a small sample of 6^th^–8^th^ grade Black youth. Mixed method data (surveys, S-L products, and focus groups) correspond directly to our conceptual model (Fig. 1).

## Method

### Setting

We collaborated with a Black/African American church (congregation and ministry 100% African American) in a Midwestern metropolitan neighborhood where unemployment rates consistently averaged 18.7% (U.S. Census, 2010), an average of 27.5% of households lived below the poverty line (Statistical Atlas, 2015), and nearly 85% of residents were African American with a median income of $23,098 (Statistical Atlas, 2015). The neighborhood was one of the city’s most violent and economically disinvested (Neighborhood Scout, 2016). The church had an active presence servicing the community via 22 ministries and promoting civic engagement and social movements. The church offered family advocacy programs, job training, financial literacy, youth and family counseling, life skills training, and anger management. After-school and summer camp programs included academic tutoring and enrichment, mentoring, creative art and recreation, and social-emotional and behavioral learning in a safe space. At study onset, the church was developing an intervention for adults exposed to violence and trauma and seeking curriculum for promoting positive youth development, mental health, and violence prevention for youth and young adults.

### Community Advisory Board

Guided by CID (Burns & Hoagwood, 2002), we invited 3 adults and 3 youth (one each from grades 6 to 8), recommended by our church partners for their commitment to youth mental health, to join our community advisory board (CAB). CAB members knew one another as part of the congregation. The CAB also included 1 FBO administrator. We emphasized authentic and meaningful youth engagement and shared decision-making between adults and youth; thus, we reminded youth each meeting to voice their suggestions, opinions, and feedback. The CAB agreed to collaborative decision-making to engage stakeholders, build consensus, and encourage creativity.

Given that youth spend most of their waking time in school, CAB youth expressed considerable interest during early meetings in using S-L to address educational inequities based on geographical, socio-economic, and racial factors. The adults agreed and wanted youth to understand *it is not about lack of achievement, but lack of opportunities to achieve*. Hence, there was high enthusiasm for the achievement gap curriculum recommended by the investigators. Further, youth strongly influenced the decision to deliver the curriculum during summer (versus fall as originally planned and preferred by the FBO administration). Youth expressed concern about school responsibilities and academics competing with attendance and suggested starting in summer may motivate participants to continue their S-L projects during fall. The CAB provided feedback on curricula (content, activities), delivery (structure, format), procedures (measures, compensation, mixed method design), and plans for interpreting and disseminating findings.

The CAB convened for a total of 6 h across three meetings and received compensation for their contributions (adults received $50 cash and youth received $50 in gift cards for each meeting, informed by previous work by Dinizulu and colleagues; e.g., Frazier et al., 2015). Feedback was also collected directly from the current CAB, and they agreed with the compensation was appropriate for the time requested for board meetings.

### Participants

Church camp staff (*n* = 3; 100% of eligible and invited staff) included two African American female full-time camp facilitators (one pursuing a Master’s degree in counseling; one with Bachelor’s degree and work experience as a public-school teacher) and one African American female program coordinator (Master’s degree in social work) who would serve as an alternate if needed. Their work experience within the past 12 months (with urban youth in community settings and in a church (e.g., “Volunteered for: an organized sport, academic or literacy program.”)) varied (range: 3–7 different types of work/volunteer experiences, M = 5.3 experiences, SD = 2.08).

Middle school youth (*n* = 21 of 26 consented, 80.7%, age range = 10 to 13, M = 11.96 years, SD = 0.906; 57% female; 100% African American), representing 18 families (3 groups of siblings) and their primary caregivers (*n* = 18), participated. Of the five youth who did not consent, two were ineligible due to significant intellectual and/or neurodevelopmental disabilities (no other inclusion/exclusion criteria). Sixty-three percent (*n* = 12) of participating families reported single-caregiver homes (100% female led). Caregivers included mothers (89%), grandmothers (5%), and fathers (5%). Two-thirds of youth (66.7%) received free or reduced-price lunch at school. Caregivers reported annual earnings below $8000 (*n* = 3, 15.7%), $20,000–$29,000 (*n* = 5, 26.3%), $30,000–$39,999 (*n* = 2, 10.5%), $40,000–$49,999 (*n* = 6, 21%), and $50,000–$59,999 (*n* = 2, 10.5%). One family did not report income. Caregivers reported completing some high school (11%), receiving a high school diploma/GED only (6%), completing some college (44%), graduating from a 2-year community/junior college (22%), and receiving a bachelor’s or advanced degree (17%).

### Procedures

#### Recruitment and Data Collection

Procedures adhered to ethical standards and were approved by the university IRB. The study corresponded to the church’s 10-week summer camp. Investigators, church leadership, and the CAB met several times to plan the study. Research staff privately met with camp staff to recruit, consent, and provide materials and training. Consented staff distributed flyers to eligible middle schoolers and their families and researchers hosted 3 “Family Nights” over 2 weeks at the church to enroll families. Caregivers and youth provided independent written informed consent and assent. Survey data were collected (via paper-and-pencil) from summer camp staff, students, and parents/guardians at the church’s community outreach center and main center of worship at baseline (during family nights), post-intervention (end of S-L program), and 3-month follow-up. To reduce burden, camp staff were randomly assigned to each report on half of the assented youth (and received $40 total). Youth received a $20 gift card and their caregiver received $15 cash at each time point.

Focus groups and interviews lasted 75 min, were facilitated by the first author (and 1 RA for groups only), and conducted in a private church room after business hours for privacy. Sessions were audio-recorded, transcribed verbatim by a HIPPA compliant and approved transcription service, de-identified, and quality checked by two RAs.

#### SMART S-L Curriculum

The Stimulating Maturity through Accelerated Readiness Training (SMART) curriculum, recommended by the NYLC (National Youth Leadership Council, 2011), was selected for its social justice efforts related to understanding and addressing the achievement gap, especially for disenfranchised youth (NYLC, 2011). SMART (see Appendix Table 1 for lessons) begins with **academic instruction** on the achievement gap (e.g., individual, family, and school factors), reflecting several decades of evidence that academic achievement protects African American youth in communities of poverty and violence (Cooley-Strickland et al., 2009); the latter half involves youth-led planning and implementation of a service project (Service-Learning Action Plan; SLAP; see Appendix Table 1) guided by local, state, and national achievement gap data. Structured **reflection** accompanies *every* session during which students examine their experiences critically, enhancing both learning and service, integrating them into a mutually reinforcing cycle to improve outcomes.

**Table 1:** Social-emotional skill building

Targeted skill	Description	Sample intervention activities
*Reach for the SCI*		
Emotion Regulation (**S**top and **C**alm Down)	Students: (1) describe what happens to their body when they are upset, (2) explain the difference between positive and negative emotions, (3) demonstrate a breathing technique, and (4) identify two options used for calming down. Skills developed: Affect Identification, Relationship between Feelings and Physiology, Relaxation, Cognitive Restructuring.	Student Handout and Discussion: *The Relation Between Feelings and Physiology*
Identify Feelings (**I** can Identify my Feelings) Problem-Solving (**I** can **I**dentify the Problem) Identifying Options and Goals (**I** can **I**dentify my Options) Identifying and Utilizing Strengths (**I** can **I**dentify my Strengths)*	Demonstrates strong feelings through a frustrating, but fun activity. It shows how people can work together for a common task and introduces the value of differences. Students identify perspectives of different actors in a problem situation, and reinforce the value in group work. Students partner up and experience the difference between options and goals in problem-solving. Students work collectively to identify individual strengths. For example, telephone game—randomly picked a letter (A–Z), thought of a personal strength starting with that letter, whispered it in the ear of the next student in line; the last student wrote the word on a poster if they heard the correct word. For Emotional Regulation row in the Description column please replace with the following: Skills developed: Affect Identification, Relationship between Feelings and Physiology, Relaxation, Cognitive Restructuring.	*Crash Landing Activity*—student is blindfolded and the partner can’t use hands. Team together to use aircraft pieces to create cups to collect rainwater in 4 min Emotion Identification Check-In: *Strawberries -N- Lemons* *What’s the Problem? Working in small groups* *Looking at Goals—*Students work together to figure out how each of them can get the most M&M candy while hands are positioned in arm wrestling form (but are not told to arm wrestle) Self-Reflection Activity: *Identifying Internal Strengths*

We made two adaptations to the SMART curriculum: (1) SMART was originally designed *with*, *by*, and *for* high school students, so we modified the language and activities for middle school students to be read, viewed, and understood more easily (see Appendix Table 1 for sample modifications); (2) we added more explicit social problem-solving (including conflict resolution), effective communication, and emotion regulation skills reflecting the most common elements of empirically supported adolescent prevention (Boustani et al., 2015) and treatment (Chorpita et al., 2005). Additionally, insight building aligns closely with three S-L Standards for Quality Practice (NYLC, 2008), specifically Reflection (e.g., examine assumptions to explore roles and responsibilities as citizens), Diversity (e.g., interpersonal skills for conflict resolution and group decision-making), and Youth Voice (e.g., knowledge and skills to enhance leadership). Infusing the curriculum with these common elements was guided by Responding in Peaceful and Positive (RiPP) Ways (Meyer & Northup, 2002) curriculum. The CAB abbreviated and simplified the problem-solving acronym (SCIDDLE) to “Reach for the SCI” (**S**top, **C**alm down, and **I**dentify emotions, problem, solutions, and strengths; pronounced “sky”), and they generated more activities for skills practice (see Table 1 and Appendix Fig. 1 for accessible pocket-sized cards for participants).

Further, other modifications were necessary to increase youth engagement and face validity, and to be responsive to teens who may be struggling academically in low-resourced and under performing schools and communities. For example, during the CAB meeting, youth and adults participated in several activities to offer feedback or recommend modifications. For instance, The Human Race activity is designed to deconstruct the achievement gap by highlighting socio-economic, racial, and cultural contributors to educational inequity (similar to a privilege walk). This activity requires diverse identities and lived experiences, but our sample was quite homogenous. We modified the activity by assigning diverse identities (e.g., White, upper class, educated) to some teens. CAB youth expressed that this activity was both important and unsettling, and their feedback informed modifications to reflection questions and race-affirming strategies (racial socialization, messages about Black pride, e.g., “I am Black and Proud”) to help study-enrolled youth cope with discomfort during the activity (e.g., Neblett et al., 2006).

#### Youth-adult Partnership Curriculum

To better support the development and sustainability of Y-APs, we also implemented a limited number of training sessions involving both youth and camp staff as guided by two Y-AP manuals (National 4-H Council, 2003; Zeldin & Collura, 2010). The CAB recommended 4 lessons to infuse within the 10-week S-L summer program (see Appendix Table 1 for description of Y-AP lessons). This training occurred prior to implementing the SMART curriculum.

#### Training, Consultation, and Fidelity

The lead investigator (first author) provided 10 h of training for the three consented camp staff. The first 6 h focused on delivering the adapted SMART achievement gap curriculum—emphasizing academic instruction and reflection related to structural barriers, inequities, and systems of oppression—and Reach for the SCI. A second 4-h staff training (2 days later) focused on building youth-adult partnerships (guided by Creating Youth-Adult Partnerships, National 4-H Council, 2003; Being Y-AP Savvy: A Primer on Creating & Sustaining Youth-Adult Partnerships, Zeldin & Collura, 2010). Active learning (e.g., modeling, role-play, practice with feedback) prepared staff to deliver the curriculum for their 6^th^–8^th^ grade youth.

The summer S-L program lasted 10 weeks (youth attended twice weekly for 90 min each). The first author was the lead facilitator for weeks 1 and 2 (camp staff co-facilitated). During weeks 3 and 4, camp staff became the primary facilitators (research staff co-facilitated). During remaining weeks, camp staff facilitated independently, with the research team observing to provide support and problem-solve as needed. Research staff provided ongoing support during 1-h weekly group consultation (*n* = 10 h, role-play and supervision) as guided by empirically supported recommendations (Edmunds et al., 2013; Salas et al., 2012).

#### 10-week Implementation

Sessions were planned twice weekly over 10 weeks (total of 20 sessions) for 90 min each. The order of a typical agenda included (a) welcome and review, (b) relaxation, (c) recreation activity (with integrated skills building), (d) didactic instruction, (e) recreation activity (with integrated skills building), and (f) wrap up. The first 4 sessions (1 to 4) emphasized youth-adult partnerships, with camp staff and youth, and shared decision-making (led by research staff only). The following four sessions (5 to 8) prioritized Reach for the SCI. The remaining sessions (9 to 20) engaged youth in SMART, guided by the National Service-Learning Cycle for Students (NYLC, 2008). The 7-step S-L cycle began with Investigation (e.g., identify needs and goals) and ended with Demonstration/Celebration and Evaluation (NYLC, 2021) during which youth presented their S-L projects to family members, peers, friends, local community members, and church administrators.

#### Measures

Mixed method data include implementation checks; staff reports (pre and post), caregiver, and youth surveys at three time points; semi-structured interviews with staff and focus groups with youth at post.

##### Implementation

Research staff observed every session and documented the following: (1) frequency and duration of S-L sessions; (2) facilitator, (3) student engagement (active participation), (4) time spent on experiential activities versus didactic instruction, (5) perceived youth and staff enthusiasm. (6) percentage of session agenda completed. (7) preparation and resources used (e.g., materials utilized for activities; space adequate or not for planned activities). and (8) open-ended statements about successes and barriers to implementation on progress notes. Attendance logs documented youths’ on time or late arrival, and early departures, to yield dosage of participation. Camp staff documented (yes or no) their program delivery after each session. Youth and research staff also reported (yes or no) on overall delivery of activities twice per week.

##### Activity Ratings

Staff rated activities each week on 7 items (1 = *Strongly Disagree* to 5 = *Strongly Agree*) designed for the study to assess enthusiasm, ease of delivery, and teen engagement*.*

##### Service-Learning Background Survey

Staff completed 27 questions about their confidence (1 = Not At All Confident to 4 Extremely Confident) discussing/teaching different topics related to S-L, planning service projects, building Y-APs, and causes of the achievement gap (e.g., racism/discrimination,).

##### Service-Learning Training Feedback Survey

Staff completed 7 satisfaction items about different aspects of the training 1 = Poor to 3 = Excellent).

##### Interviews and Focus Groups

Focus group guides for youth explored time and activity expectations, relevance of knowledge and skills gained, competing priorities (e.g., after-school tutoring, family obligations), and overall satisfaction. Semi-structured interviews for staff assessed alignment of the S-L curriculum with camp goals and activities (e.g., How well does the curriculum resemble other summer activities?) and self-efficacy (e.g., Which activities were you best equipped to facilitate and why?). The interview with the church program coordinator explored unique experiences, adequacy of resources, barriers (e.g., unpaid planning time), and facilitating factors for training, supervision, and curriculum delivery.

#### Direct Targets of Intervention (Proximal Youth Outcomes)

##### Community Belongingness

Youth completed the Psychological Sense of School Membership Scale (Goodenow, 1993; 18 items, 6-point Likert scale, 0 = not at all true of me to 5 = very true of me; *α* = 0.80, e.g., “I feel like a real part of this community,” “I can really be myself in this community”). Items were summed to create a single composite score (*α* = 0.89).

##### Social Responsibility

Youth completed Greenberger and colleagues’ (1975) Social Responsibility scale (11 items, *α* = 0.90, 4-point Likert scale 1 = strongly disagree to 4 = strongly agree; high score indicates a high level of social responsibility, e.g., “It’s really not my problem if my neighbors are in trouble and need help”). Two (5/6^th^ grade) and one (7/8^th^) negatively worded items were reverse coded. Younger youth completed the version for 5/6^th^ grade (*α* = 0.80) and older youth completed the version for 7^th^/8^th^ grade (*α* = 0.33). Items were summed to create a single composite score. Analyses were run using both raw scores and scores standardized within each age group. There was no difference in results; analyses with raw scores are presented.

#### Youth Academic, Social, Emotional, and Behavioral Adjustment (Distal Outcomes)

##### Academic Motivation

Youth completed the General Academic Motivation Subscale of the Children’s Academic Intrinsic Motivation Inventory (CAIMI; Gottfried, 1986; 18 items on a 1 to 5 Likert scale; *α* = 0.69–0.75). Items represent valuing schoolwork, general school motivation, academic values, and effort; they are summed to create a single composite score (*α* = 0.69).

##### Mental Health Screener

Youth completed the Strengths and Difficulties Questionnaire (SDQ, age 11–16; Goodman, 1997; 25 items; *α* = 0.84; Likert scale 0 = “not true,” 1 = “somewhat true,” and 2 = “certainly true”) that yields five subscale scores (5 items each): emotional symptoms, conduct problems, hyperactivity/inattention, peer relationship problems, and prosocial behavior. Items within each subscale were summed to create composite scores.

##### Social Competence, Emotional, and Behavioral Functioning

The Social Skills Improvement System (SSIS; Gresham & Elliott, 2008). Parents and staff reported youth social skills (46 items) and problem behaviors (33 parent items, 30 staff items) along a 4-point scale (*never* to *always*). Our sample yielded high baseline reliability for both parent and staff report of Social Skills (*α* = 0.97 and 0.95, respectively) and Internalizing (*α* = 0.95) and Externalizing (*α* = 0.93).

### Analytic Plan

#### Quantitative Data

We present frequency, duration, and coverage of material (feasibility), perceptions of activities (acceptability), extent to which staff attended trainings, consultation and S-L sessions (engagement), and extent to which they presented planned content (adherence) and youth completed assignments and activities (dosage). Findings were interpreted with stakeholders. Influence on youth outcomes measured longitudinally was tested using Linear Mixed-Effects Models in SPSS. All models included youth, gender, and age at baseline as fixed effects, with time modeled as a repeated factor. For parent and youth report of outcomes, time had three levels: baseline, post-test, and follow-up. For S-L staff reports of outcomes, time included baseline and post-test reports only. Models used restricted maximum likelihood estimation with an unstructured covariance matrix between time points. The mixed-effects approach allowed us to retain all *n* = 21 youth in analyses, including youth missing data at post-test (*n* = 2) or follow-up (*n* = 6).

#### Qualitative Data

Acceptability was also assessed using thematic analysis (e.g., Braun & Clarke, 2006). Transcripts from interviews and focus groups were reviewed several times independently by four members of the research team to generate codes deductively. These codes were then applied independently by the same four coders to each transcript. Coders met to review transcripts when Kappa was below 0.80 and discrepancies were discussed and resolved (Guest & MacQueen, 2008). During discussions, patterns in the data were identified, while accounting for key points relevant to research questions, to generate themes. Dedoose 7 was used for data analysis (Dedoose, 2021).

## Results

### Mental Health Need

Teens (*n* = 21) reported, at baseline, significant mental health concerns as measured by the SDQ broadband standardized scales: Total Difficulty (borderline 19%; abnormal 76%), Emotional Problems (borderline 28.6%; abnormal 71.4%), Conduct Problems (borderline 43%; abnormal 19%), Peer Problems (borderline 38%; abnormal 29%), and Prosocial (borderline 24%; abnormal 19%).

### Feasibility

#### Staff Engagement and Adherence

Prior to training all three staff reported feeling Very Confident in their knowledge about the goals of S-L and Somewhat to Extremely Confident about their ability to support youth to take social action, reflect on their S-L experiences, cope with prejudice or discrimination, explore problems in their community, and plan a community service project to address the problem. All camp staff reported that “training as a whole” was Excellent and “helped them become familiar with the requirements of being a S-L instructor” was Satisfactory to Excellent.

Consented camp staff (*n* = 3, includes the alternate) attended both trainings (10 h total over 2 days) and facilitated or co-facilitated 100% of the first seven S-L sessions. At least one staff member facilitated 16 sessions (80%, remaining 4 sessions delivered by research staff). One staff member attended 9 of 10 consultations, and one attended 8, for ongoing implementation support.

Research staff live-coded adherence for all 14 sessions (co)facilitated by camp staff (camp staff also completed 7 (of 14) session checklists). Youth reports were inconsistent and not reliable (discrepancies documented by research staff) possibly due to social desirability or teens not understanding the instructions of how to complete the checklists. Overall, camp staff strongly endorsed close adherence to the S-L protocol (see Table 2): general session procedures (91%), S-L project planning (100%), S-L based activities and instruction (78%), and process (66.2%).

**Table 2:** Fidelity checklist by session facilitated by S-L staff: project staff report

Week:	3	4	4	5	6	6	7	7	8	8	9	9		
Day:	2	1	2	1	1	2	1	2	1	2	1	2	Fidelity^c^	%^c^
***General procedures***														
Welcomed youth and reviewed agenda	✓	✓	✓	✓	✓	✓	✓	✓	✓	✓	?	✓	11/11	100.0
Did a team building exercise	No	✓	n/a	✓	✓	No	✓	✓	✓	✓	✓	No	8/11	72.7
Reviewed material from previous session	✓	✓	?	✓	✓	✓	✓	No	✓	✓	✓	✓	10/11	90.9
Did strawberries & lemons check-in	✓	✓	✓	✓	✓	✓	No	✓	✓	✓	✓	✓	11/12	91.7
Gave overview of topic and discussed why it was important	✓	✓	✓	✓	✓	✓	✓	✓	✓	✓	n/a	n/a	10/10	100.0
Taught students a specific skill	✓	✓	✓	✓	✓	✓	✓	✓	✓	No	✓	✓	11/12	91.7
Used handouts to talk about the day’s topic	✓	✓	✓	✓	✓	✓	✓	✓	✓	✓	✓	✓	12/12	100.0
***Service-learning based activities***^***a***^														
Led 1^st^ program activity	✓	✓	✓	✓	✓	✓	n/a	n/a	n/a	n/a	✓	No	7/8	87.5
Led 2^nd^ program activity	✓	✓	✓	No	/	n/a	n/a	n/a	n/a	n/a	n/a	n/a	4/5	80.0
Led 3^rd^ program activity	✓	✓	n/a	No	n/a	n/a	n/a	n/a	n/a	n/a	n/a	n/a	2/3	66.7
Led 4^th^ program activity	n/a	n/a	n/a	n/a	n/a	n/a	n/a	n/a	n/a	n/a	n/a	n/a	n/a	n/a
***Service-learning action-planning (SLAP) activities***^***bc***^														
Helped students with 1^st^ SLAP goal	n/a	n/a	n/a	n/a	n/a	n/a	✓	✓	✓	✓	✓	✓	6/6	100.0
Helped with 2^nd^ SLAP goal	n/a	n/a	n/a	n/a	n/a	n/a	/	/	n/a	/	n/a	/	4/4	100.0
Helped with 3^rd^ SLAP goal	n/a	n/a	n/a	n/a	n/a	n/a	n/a	n/a	n/a	n/a	n/a	/	1/1	100.0
Helped with 4^th^ SLAP goal	n/a	n/a	n/a	n/a	n/a	n/a	n/a	n/a	n/a	n/a	n/a		1/1	100.0
***Process-related items***														
Gave students time to work on personal reflection	✓	✓	✓	No	No	No	?	✓	No	✓	No	No	5/11	45.5
Allowed for group discussion of personal reflection	✓	✓	✓	✓	✓	✓	No	✓	✓	✓	✓	✓	11/12	91.7
Invited youth to give ideas/opinions	✓	✓	No	No	✓	✓	✓	✓	✓	No	✓	✓	9/12	75.0
Planned goals with youth for next session	No	No	?	No	No	No	No	No	✓	✓	✓	No	3/11	27.3
Allowed students to co-lead activities	✓	✓	✓	✓	✓	✓	✓	No	✓	✓	✓	✓	11/12	91.7

Weeks 1, 2, and 3 session 1 was Y-AP training facilitated by research staff only—fidelity report not required. A staff fidelity report was not filled out for week 5, day 2. Week 10, fidelity report not required due to demonstration and celebration of S-L project

Key: ✓ = occurred during session. No = did not occur during session. n/a = was not part of the planned session curriculum. ? = missing data

^a^The number of activities in each week varied from 1 to 4

^b^SLAP activities did not begin until week 7

^c^Calculations exclude weeks with missing data and/or where a given event was not part of the planned session curriculum

#### Youth Dosage

Teen attendance across 20 sessions averaged 79% (16 of 21 assented teens; SD = 3.5; range 9–20 teens). Competing church or summer school classes resulted in 38% (*n* = 8) arriving late and 14% (*n* = 3) leaving early (different teens from late arrivals). Sessions (planned for 90 min) lasted an average of 86 min (SD = 8.08, range = 73–97 min). Half began on time, and the other half averaged 6 min late (range = 0–17 min). Seventy-five percent of sessions ended on time.

#### Service Project: Students Expressing and Addressing Inequities

For the Demonstration and Celebration (last step of the S-L cycle), teens shared causes of the “achievement gap” (renamed “opportunity gap”) from a social injustice and systematic oppression lens. They were disappointed to learn that most of their schools (K–8^th^) were on the lower end of the opportunity gap. S-L participants introduced three service projects titled (a) The Lending Library, (b) Guest Readers—to address the 30-million-word gap (Hart & Risley, 2003), they learned about in their research, and (c) Tutoring Services.

### Acceptability

Camp staff completed 7 of 10 weekly activity ratings (1 = *Strongly Disagree* to 5 = *Strongly Agree*), which indicated “students were actively engaged (M = 3.7, SD = 0.80, range = 3.0–4.5), camp staff were enthusiastic (M = 4.07, SD = 0.67, range = 3.0–4.5), activities were executed with ease (M = 3.30, SD = 0.81, range = 2.0–3.5), academic content was easy to teach (M = 3.92, SD = 0.98, range = 2.0–4.5), space was conducive (M = 4, SD = 0.96, range = 2.0–4.5), materials were adequate (M = 4.35, SD = 0.38, range = 4.0–5.0), and discussion went well (M = 3.90, SD = 0.75 range = 3.0–4.5).

For the focus groups, sixteen of 21 youth were randomly invited to focus groups and 15 agreed to participate (one declined due to living too far from the church and was replaced by another randomly selected student). Two groups were completed (*n* = 6 and *n* = 3) within 1-month from post-test. Seven youth did not participate due to the following: a no show (*n* = 2) or cancelled due to school obligations (*n* = 3), living too far after moving (*n* = 1), or illness (*n* = 1).

Six themes emerged from youth focus groups. Theme 1, *Value and Inclusion*, reflects perceptions that explicit attention to Y-AP helped youth feel included and heard, and expectations for them to lead were perceived as new in relation to issues and events in their community and city. For instance, “sometimes kids are not included in things with the city or them things and I think that it was good because we got to share our ideas about how we feel about things inside the community that we don’t usually get to do because we are kids and sometimes our ideas don’t matter” (Female 1). Theme 2, *Shaping Personal and Societal Differences*, reflects elevated awareness among youth of societal and racial inequities, which motivated interest in knowing more, working hard, and teaching others. To this end, one focus group participant (Male 1) stated, “With the knowledge I gained […] one of the things is like when we didn’t have all the resources, it just made me feel like, because we don’t have any resources, I gotta make the best of these resources. So that made me study harder, focus more, and now I’m doing better in school than I was last year and the years before.” Theme 3, *Knowledge, Skills, and Strategies*, reflected youth appreciation for learning how to build confidence, resilience, and resourcefulness. In particular, youth explicitly connected S-L to their daily lives, and Reach for the SCI was described as lifting self-confidence and coping with being angry or frustrated: “if you get upset, you count one to five, one, two, three, four, or breathe in and out” (Female 1). Theme 4, *Personal Development and Self-Efficacy*, highlighted that collaborating with adults and using their voices to lead on important issues boosted confidence, motivated youth to educate adults about Reach for the SCI skills, and empowered them to advocate more for themselves in other settings. Many noted the culminating activity as particularly powerful, one participant describing that these feelings came from when “I was up on stage and I was telling them like to use SCI and stuff. Yeah, so I told my family, I told like Facebook about the achievement gap” (Female 2). Theme 5 was *“Fun” Promotes Engagement*, suggesting that dynamic and high-energy activities involving movement and competitive games were perceived as the most engaging and most memorable, enhancing youths’ overall investment and learning: “Because after [the human knot], I think everybody communicated more after the human knot. At first I wasn’t talking to a lot of people before the human knot and after that, I just started being myself, like how I be at school” (Male 2). Theme 6, *Positive Experiences*, reflected youths’ high enthusiasm for the program, preference for particular activities (e.g., Jeopardy) that were most enjoyable and influential, and overall takeaways that were useful for their lives and into the future: “I could use [what I learned] in the future…if I want to be, like if my dream is to be a teacher, lawyer, anything that we can do with the community. That will be easy because I’m talking about just because of the race, just because the color of your skin, it don’t mean that you’re smarter than others” (Female 1). Each theme emerged several times throughout the conversations with the youth. To demonstrate the frequency of each and provide depth, Table 3 provides greater detail and illustrative quotes.

**Table 3:** Themes from staff semi-structured interviews and youth focus groups

Theme	Frequency	Examples	Example quote
***Staff***			
Tools for Successful Implementation	25	Training and consultation; More staff; Cheat sheets; Better quality of youth-adult partnerships; Demonstration and celebration of service-learning projects	“I thought that was good because it’s a good time for us to discuss. I like it because it felt collaborative … I felt like it was an open door for us to think about other things that we could do better.” (Female 1)
Shaping Perspectives of Societal Differences	23	SMART: Solutions to Addressing the Achievement Gap (Understanding and addressing racial disparities) Researching data about youth’s own school academic statistics and resources to address the achievement gap	“Yeah and the academic as well because they were able to see those numbers where we’re struggling at…I think they were able to come up with an actual service-learning plan…to fix some of those specific issues.” (Female 1)
Valuing and Including Youth	16	Promoting Youth Voice – Developing a Common Vision; Planning Demonstration & Celebration Activities (e.g., Writing letter to business owners/mangers requesting donations for food and school supplies); Community service projects	“I’ve always been for youth-adult partnerships. I’ve always felt like youth have something to contribute, we need to listen, we need to let them be more involved…” (Female 2)
Challenges to Implementation	13	Multiple roles; Not feeling prepared; Too many changes; No prep time built in; Slow to start; Kids knowing each other	“There was turnover happening all at the same time. We lost a supervisor all at the same time. So people were just filling in the blanks.” (Female 1)
Coordinating Systems	11	More involvement from administration; Improved communication between all staff and administration; Support from parents	“I feel like having support from parents and administration is important…I think the kids when they see that we’re all making a big deal of it, I feel like then they’ll come and there’s an expectation to be a part of this now. (Female 1)
Tools for Positive Youth Development	12	Trust Fall; Reach for the SCI – Emotional Regulation and Problem-Solving Curriculum; Strawberries N’ Lemons	“Reach for the SCI kind of teaches them to be more in touch and in tune with themselves, but also how to work with others…problem solving together…”(Female 1)
***Youth***		Intervention activity	
Value and Inclusion	9	Check-In (Strawberries & Lemons) Team Building Exercises (Marshmallow activity)	“We had all used teamwork…they were encouraging us to work.” (Female 2)
Shaping Perspectives on Personal and Societal Differences	47	The Human Race	“Just to know certain things that are going on because of different kind of race…So, like, black people, we’ve got the lowest percentage against whites… Some people might feel disrespected or, you know, yeah.” (Female 4)
Applying Knowledge, Skills, and Strategies	50	Reach for the SCI	“I learned to control your emotions.” (Female 4)
Personal Development and Self-Efficacy	35	Demonstration/Celebration Community Service Project (CSP) Presentation	“I have learned to have confidence in yourself.” (Male 1)
“Fun” Promotes Engagement	23	Jeopardy	“More things that are interesting like something that we are learning but make it fun. Like we could play some stuff like sports as an activity.” (Male 2)
Program Satisfaction	13	Demonstration/Celebration CSP Presentation	“I think it was useful because some of us may not have knew so it probably was useful.” (Female 3)

Six themes emerged from camp staff interviews (supplemented by progress notes), some mirroring youth perspectives and others relating to program development. Theme 1 *Tools for Successful Implementation*. Weekly consultation was perceived as integral for answering questions, clarifying purpose of activities, problem-solving, and boosting confidence. Camp staff reported using four tools consistently to manage disruptive behavior by a few youth: redirection (e.g., “Clap your hands if you hear me.”), reminder of group rules (e.g., put cellphones in basket), and small group work to encourage engagement, enhance leadership, and decrease interruptions. Theme 2 *Challenges to Implementation* was described as both systemic and personal, including inadequate time for planning lessons and preparing activities, lack of confidence facilitating sessions and connecting with youth, and disruptive behaviors by a few youth with emotional/behavior dysregulation difficulties (documented in 15/20 sessions, e.g., inattention/ disengagement, overuse of cell phones, noncompliance with general rules or specific directions). Theme 3 was *Valuing and Including Youth* where Y-AP were noted as useful in promoting new program structures for team building, generating new dynamics between staff and youth, and facilitating genuine connections and increased youth engagement. For example, “I think that was very helpful for them and then not just trying to take over like look it's what we say and trying to get them to add more input. I think that was very helpful too, recognizing that I can't do it without you and I think that some of them were able to see that and so I felt like they were more involved because of that” (Staff 1). Theme 4 *Coordinating Systems* described challenges related to commitment, coordination, and communication across systems and stakeholders (students, staff, administrators, and families). Specifically, staff wanted more clarity about expectations, more support from administration, and more effort to engage parents: “I definitely feel like if they could have seen more of an administrative presence they would have taken it more seriously, especially because a lot of the campers are members at this church or their parents work for the outreach center” (Staff 2). Theme 5 *Tools for Positive Youth Development included* staff embracing activities for building team and community (e.g., trust fall), solving concrete problems, fostering social-emotional growth (e.g., Reach for the SCI), and overall empowering youth (e.g., Demonstration and Celebration). Some staff highlighted the order of these activities as creating a clear pathway for students to learn and take action: “So it's like you see a reason to problem solve, but then you also learn team building at the same time so you get this consistency, but then you also see there's a problem out here, guess what, since we’ve been working together, did you know that we could actually work on this together as well” (Staff 1). Theme 6 *Shaping Perspectives of Societal Differences* included exploration of youth critical consciousness about the achievement gap and other societal issues. While questions of inequity in relation to violence and achievement were not experientially new to youth, the staff saw unique value to the explicit instruction about the achievement gap and interactive learning: “I feel like when we discussed achievement gap it kind of brought it together for them, like, oh, we’ve got real problems out here because really there was some emotion coming out of some of these. Yeah, so they could really see that this is a real issue. We don’t know what to do about it, but we do not like what we’re seeing when we’re looking at these facts and these numbers” (Staff 2). Illustrative quotes and frequency counts are provided in Table 3.

### Promise

Appendix Table 2 shows the Type III tests of fixed effects for age, gender, and time from all mixed-effects models. For youth self-report, there were significant effects of time for Emotional Symptoms, Peer Problems, and Prosocial Behaviors, but not for youth self-reports of Conduct Problems, Academic Motivation, Community Belongingness, or Social Responsibility. Time was not a significant predictor for the three parent report outcomes. There was a significant effect of time for staff report of Internalizing Problems but not Externalizing Problems or Social Skills.

The estimated marginal means for each time point, for each outcome, are presented in Table 4. Pairwise comparisons revealed that post-test and follow-up scores were significantly lower than baseline for youth report of Emotional Symptoms, and Peer Problems, and youth prosocial scores were significantly higher at post-test and follow-up than at baseline. There was no difference in scores between post-test and follow-up for any outcome. For staff report, post-test internalizing scores were significantly lower than baseline scores.

**Table 4:** Estimated marginal means^a^ for outcomes at each time point

	Baseline (BL)			Post-Test (PT)			Follow-up (FU)^b^			
Outcome	*N*	Mean	SE	*N*	Mean	SE	*N*	Mean	SE	Contrasts^c^
***Youth self-report***										
Community Belongingness	21	62.61	3.08	19	61.50	2.88	15	62.10	3.66	
Social Responsibility	20	27.71	1.23	19	26.46	1.15	15	25.68	1.04	
Academic Motivation	20	64.75	1.84	19	62.82	2.25	15	59.57	2.16	
Emotional Symptoms	21	6.10	0.32	19	3.60	0.60	15	3.78	0.75	PT, FU < BL
Conduct Problems	21	3.21	0.37	19	2.63	0.53	15	2.54	0.60	
Peer Problems	21	4.04	0.21	19	3.02	0.43	15	2.55	0.46	PT, FU < BL
Prosocial Behavior	21	5.60	0.28	19	6.93	0.49	15	7.60	0.54	PT, FU > BL
***Parent report***										
Social Skills	21	85.73	4.30	19	91.17	4.74	15	84.70	4.26	
Externalizing	21	8.06	1.25	19	8.70	1.23	15	8.01	1.60	
Internalizing	21	6.22	0.97	19	6.63	1.15	15	7.04	1.35	
***Staff report***										
Social Skills	21	85.38	4.02	19	89.78	3.41	–	–	–	
Externalizing	21	7.49	1.22	19	7.16	1.63	–	–	–	
Internalizing	21	3.82	0.82	19	2.77	0.70	–	–	–	PT < BL

^a^Means adjusted for youth age and gender

^b^Staff reports of youth outcomes were not obtained at follow-up

Contrasts are significant *p* < .05

## Discussion

The current study leveraged the capacity of Black churches to promote mental health for middle school youth through S-L programming. We collaboratively adapted and piloted a S-L program centered in social justice to address the achievement gap. We also infused empirically supported recommendations for social problem-solving and emotional regulation to directly target behavioral, social, and emotional outcomes. Mixed method findings revealed high feasibility and acceptability, evidenced by consistent attendance and engagement by staff and youth; high adherence, evidenced by completion of planned sessions, consultations, and training; and reports by staff and youth that S-L facilitated skills, shaped perspectives, inspired openness, and created a space for all to feel valued and included. Constructive feedback will inform the next iteration of the program. Three time points of surveys completed by youth, and two time points completed by staff revealed reductions in emotional and behavioral problems and improvement in social functioning, across time, by reporter, for specific subgroups of participating adolescents.

### Cultivated Social Justice Inspired Service-learning in a Black Church

The S-L curriculum aligned well with the goals and values of the church and fit relatively seamlessly into summer programming. All 20 S-L sessions were delivered as planned including content, instruction, and activities facilitated mostly by camp staff, who also completed all trainings and took full advantage of ongoing consultation. Attendance and adherence exceeded the average rates of retention and completion in traditional mental health services for urban, low-income youth (McKay et al., 2005). Strong adherence reporting by research staff highlights that organizations can confidently rely on internal monitoring procedures to inform ongoing quality improvement.

High engagement and enthusiasm among adolescents and camp staff illustrate that youth felt valued, staff felt confident, and content felt meaningful. Youth especially enjoyed physically active sessions that raised critical awareness of structural and historical inequities in their communities and daily lives. Camp staff echoed these points and appreciated tools to promote youth engagement, social-emotional learning, and positive development, and content to shape their perspectives on societal differences. Findings align with several S-L Quality Standards (NYLC, 2008), including Meaningful Service, Youth Voice, Link to Curriculum, Diversity, and Progress Monitoring.

### Service-learning Holds Promise to Promote Mental Health

The S-L program demonstrated effects on some distal but not proximal outcomes based on adolescent self and camp staff report. Overall, adolescents reported fewer Emotional Symptoms and Peer Problems, and more prosocial behaviors, after completing the program, and gains were maintained at 3-month follow-up. Camp staff also reported fewer Internalizing Behaviors among youth following completion of the program. Parent reports revealed no changes over time for adolescents’ social, emotional, and behavioral functioning, which may reflect that more time, duration, or depth may be needed for improvements to generalize to observable behaviors at home.

In contrast to the qualitative data, we saw no improvement in the proximal outcomes in Community Belongingness and Social Responsibility. Given that the oppressive social climate can deeply impact their communities and their own civic development (e.g., Hope et al., 2015), we have considered that the measures herein of Social Responsibility and Community Belongingness (designed with WHAT samples in mind) may have failed to capture the nuance and salience of these constructs in the lives of Black youth. There are efforts ongoing to develop measures for Black adolescents and emerging adults (e.g., Hope et al., 2019). Despite this disconnect, focus group data and themes from this sample present potential insight to include when conceptualizing social responsibility and community belongingness for this population.

Focus groups with youth and interviews with camp staff together revealed themes related to proximal and distal outcomes in the conceptual model. Specifically, youth expressed the importance of feeling valued and included and described elevated awareness (psychological engagement) about social and racial inequities that inspired their sense of social responsibility and community belongingness. Youth explicitly discussed applying what they learned and feeling more self-confident, increased self-efficacy, and ability to advocate for themselves. Interviews with staff also supported the conceptual model, as they described how the program provided ways to value and engage youth, and tools for positive youth development, and that with these they observed growing competencies in the areas of problem-solving and social-emotional wellness.

### Lessons Learned: Facilitators and Barriers

Several facilitating factors (see Damschroder et al.’s Consolidated Framework for Implementation Research, 2009) appeared to increase feasibility of implementation: (a) *trialability and modifiability:* staff valued opportunities to experiment with key features of curriculum on a small-scale, build experience and expertise, and reflect and receive feedback. CAB input influenced Reach for the SCI (adapted from RiPP curriculum) and The Human Race (activity adapted from SMART); (b) *compatibility:* (i.e., tangible fit between meaning and values of the organization and how the intervention fits with existing workflows and systems). Administrators and staff indicated that S-L aligned with their faith-based values, met their annual goals of providing community service, civic engagement, and mental health and violence prevention programming, and fit within their existing summer programming and workflow; (c) *readiness for implementation*: evidenced by S-L staff engagement in training and consultation, accountable leadership, high motivation and enthusiasm, consistency and adherence, and positive attitudes and involvement; (d) *reflecting* and *evaluating:* staff and youth provided extensive feedback about progress and quality of experience, promoting shared learning and iterative improvements for sustained implementation. 

The most significant and consistently reported barriers to implementation were poor coordination of systems and communication between administrators and staff (exacerbated by high turnover) and inadequate engagement or support by parents and caregivers. The literature points to the importance of high-quality communication across organizational stakeholders for effective program implementation, specifically peer collaboration, open feedback and review among peers and across hierarchical levels, cohesion between staff, and informal communication quality (e.g., Glisson et al., 2008). Staff perceived that parents and administrators could do more to increase youth attendance, aligning with evidence that parent involvement and communication are important for youth education and outcomes (Barnard, 2004). High turnover contributed to staff feeling mentally and physically drained by fulfilling multiple roles, supporting inexperienced staff, and substituting for absent colleagues or supervisors, all of which reduced their time for planning and preparing activities. Weekly consultations allowed research staff to empathize and problem-solve (e.g., carve out prep time, create activity cheat sheets) with camp staff.

### Limitations

The small sample and open trial design suited the pilot nature of this work, but findings should be interpreted with consideration for limitations. First, dosage was 20 sessions over 10 weeks, which may not be adequate to influence or sustain academic outcomes. The literature shows a strong correlation between duration and standards being implemented (e.g., Shek et al., 2021). Program delivery during summer may also have interfered with connecting content to academic engagement. In the next iteration, we plan to begin S-L in the summer and continue after school during fall, allowing for more time to implement the full recursive experiential learning cycle. This process empowers youth to take ownership by developing and engaging in new projects over time, enhancing the benefits for themselves and their communities. Notably, research staff documented difficulties with sustaining equitable Y-AP; sometimes camp staff and youth unintentionally fell back into traditional roles of teachers leading and students following, needing encouragement and reminders to lead and lean into decision-making. Extending time beyond 10 weeks may help deepen and sustain behaviors and thought processes for equitable relationships. Further, managing youth with significant externalizing behaviors was not discussed at training with S-L staff. It is recommended to practice strategies prior to implementation. Despite limitations, reliable improvement for individual youth at three levels of mental health need (i.e., social, emotional, and behavioral) is encouraging and suggests the model holds promise for strengthening social skills and reducing problem behaviors.

## Conclusion

This study advances a robust literature on the important role of Black churches in promoting civic engagement and health promotion in the context of our nation’s systemic oppression. The current study advances that literature as the first (to our knowledge) to examine the infusion and outcomes of a church S-L program to promote Black adolescent mental health. Participating youth demonstrated deep concern related to causes and effects of the achievement gap (“opportunity gap”), reflecting their increased knowledge of how systemic injustice limits access to educational opportunities and community resources, altogether disrupting pathways to well-being. The Black church represents a critical physical and social space for youth to address structural inequities, especially through S-L. This study presents encouraging data about iterative, mixed method, and community-engaged models of socially responsive research that can optimize mental health, educational equity, and lift every voice among Black youth.

### Supplementary Information

Below is the link to the electronic supplementary material.

Supplementary file1 (DOCX 36 KB)

## Data Availability

Data supporting tables 2-4 and supplementary appendix table 2 are not publicly available in order to protect participant privacy. These datasets can be accessed on request from Sonya Mathies Dinizulu, Department of Psychiatry and Behavioral Neuroscience, University of Chicago Medicine, Chicago IL. Email: sdinizulu@uchicago.edu, upon the completion of a Data Usage Agreement.
