# Supplementary material for: Lift Every Voice: Engaging Black Adolescents in Social Justice Service-Learning to Promote Mental Health and Educational Equity
Source: Prev Sci. 2023 Sep 15;25(1):68–84. doi: 10.1007/s11121-023-01570-w (PMC10830586; doi:10.1007/s11121-023-01570-w)
Supplement: Supplementary file 1 — Supplementary file1 (DOCX 36 KB) [file 11121_2023_1570_MOESM1_ESM.docx]

Appendix Table 1: Description and Modifications of Program Components

| **Schedule** (W=week D=day) | **Program Component** | **Primary Facilitators** (Camp Staff vs. Research Team) | **Modifications** |
| --- | --- | --- | --- |
| Youth Adult Partnerships (Y-AP) | | | |
| W1D1 | Focus: Y-AP Team Building  Activities: ‘Establishing Group Norms’; ‘The Team Game’ | Research Team | -Collaboratively developed group norms/rules through a “healthy community” lens aided by **youth leaders** who recorded rules on a flip chart.  -Exercise was a team-building game to enhance camaraderie and the comfort level among participants. Each group presented its team name, three traits in common, and a cheer. *No changes were made*. |
| W1D2 | Focus: Vision and Barriers to Y-AP  Activities: ‘Developing a Common Vision’; ‘Identifying Barriers to Effective Partnerships’ | Research Team | -Posted the “vision” and “barrier” questions and themes from responses on a flip chart, which was helpful to participants who were visual learners; method also served to discuss where Y-APs currently exist (or can exist) within their program/organization; rotated through partners to discuss written responses only 2-3 times before coming together as a larger group. |
| W2D1 | Focus: Talking It Over and Review of Youth Perceptions of Adults  Activities: ‘Talking it over: Learning to Give and Receive Feedback’; ‘P.A.R.T.N.E.R  Activity: Evaluating the Effectiveness of Y-AP’ | Research Team | -Discussed the value of giving and receiving feedback to improve Y-APs; provided pre-written scenarios around conflict resolution for the groups to reflect on how (i.e., action plan) both parties can contribute to positive outcomes.  -Cut questions down to five from the P.A.R.T.N.E.R handout which facilitated a check-in on the effectiveness of the group’s work for that day and to reflect on the quality of the current partnership relationships. |
| W2D2 | Focus: Identify leadership Styles and Knowing Yourself  Activities: ‘Attributes of a Leader’; ‘Where Are You Coming From? Looking at Shared Leadership’ | Research Team | -Edited the terminology on the handouts to better fit the reading and developmental level of participants.  -Discussed prominent leaders in the Black community and their attributes. |
| Reach for the SCI: Stop, Calm Down, & Identify *(adapted from Responding in Peaceful & Positive Ways [RiPP])* | | | |
| W3D1 | Focus: Module 1– Stop and Calm Down  Activities: ‘Mindfulness Activity’; ‘Self-talk’ Activity; ‘Breathwork’ | Co-facilitate | -Created a visually-appealing Reach for the SCI bookmark to reinforce the lesson.  -Led a discussion following relaxation activities that participants could easily grasp in order to aid them in identifying their own physical signals of anger and anxiety.  -Edited handouts (e.g., format, font, visuals, less dense) tailored towards age/grade level to make content about feelings & physiology easier to integrate. |
| W3D2 | Focus: Modules 2-3– Identifying Feeling/Problem  Activities: ‘Crash Landing Activity’ | Co-facilitate | -Edited the terminology on the handout/activity to better fit the reading and developmental level of participants in order to demonstrate strong feelings while working together on a common task. |
| W4D1 | Focus: Modules 4-5– Identifying My Options/Goals + Identifying and Utilizing My Strengths  Activities: ‘M&M Activity’; ‘Mapping Activity’ | Co-facilitate | -‘M&M Activity’: Used relevant examples (e.g., specific to Chicago) when talking about conflict, options, and consequences.  -‘Mapping Activity’: Simplified handout to identify strengths and resources across multiple ecological levels (e.g., individual, family, school). |
| W4D2 | Focus: Review of previous lessons | Co-facilitate | NA |
| SMART: Youth Solutions to the Achievement Gap | | | |
| W5D1 | Focus: Understanding the Achievement Gap  Activities: ‘Human Race Activity’; ‘Deconstructing the Gap’  Review of Constructs: Racism, Systematic Oppression, Structural Inequities, etc. | Camp Staff | -‘Human Race Activity’: Because the cohort was homogenous, profiles of different demographics were created for youth to assume during the activity.  -Deconstructing the Gap**:** Revised ‘What’s Causing the Gap?’ handout to make grade appropriate (e.g., visuals, videos, made material less visually dense).  -  Implemented cultural socialization (a form of racial-ethnic socialization) strategies to affirm Black pride, culture, and significant contributions to history. |
| W6D1 | Focus: Exploring the Link—the Individual and Academic Achievement  Activities: ‘Circle of Success’; ‘Mapping Success | Camp Staff | -Edited handouts (e.g., format, font, visuals, less dense) tailored towards age/grade level to make content easier to grasp.  -Facilitated a discussion around ‘-isms’ (e.g., racism, classism) and related it to Black Americans.  -‘Circle of Success’: Revised “What is Success?’ questions to better fit the reading and developmental level of participants. |
| W6D2 | Focus: Exploring the Link—Home/Family Background and Academic  Activities: Trivia (Team Building); ‘Do Words Really Matter?’ ‘Do Teachers Really Matter?’ ‘Expecting Excellence’ | Camp Staff | -Edited handouts (e.g., format, font, visuals, less dense) tailored towards age/grade level to make content easier to grasp.  -’Do words really matter’ English test: chose a developmentally appropriate poem/reading piece |
| W7D1 | Focus: Exploring the Link—Teachers and Academic Achievement  Activity: ‘Marshmallow Activity’; ‘Math Test Simulation’ | Camp Staff | -Edited handouts (e.g., format, font, visuals, less dense) tailored towards age/grade level to make content easier to grasp.  -‘Math Test Simulation’: revised to make grade appropriate. |
| W7D2 | Focus: Exploring the Link—Schools and Academic Achievement  Activity: Trust Fall (Team Building); Math Test Simulation | Camp Staff | -Edited handouts (e.g., format, font, visuals, less dense) tailored towards age/grade level to make content easier to grasp.  -‘Math Test Simulation’: revised to make grade appropriate. |
| W8D1 | Focus: SLAP Sections A, B, C  Activity: Jeopardy (Team Building) | Camp Staff | -Jeopardy included simple questions about content from Y-AP, Reach for the SCI, & Academic Achievement Gap materials.  -(A) Organization information→ Group name, members’ names, adult mentors assisting with the project. *No changes were made*.  -(B) School Research→ provided laptops to groups so that participants could do their own research (e.g., what are the racial/ethnic and socioeconomic demographics) for their school. |
| W8D2 | Focus: SLAP Sections B, E, F  Activity: Stand up/Sit down (Team Building) | Camp Staff | -Edited handouts (e.g., format, font, visuals, less dense) tailored towards age/grade level to make content easier to grasp.  -(F) Goal-setting→ Aided students in developing SMART (specific, measurable, appropriate, realistic, and tangible) goals. |
| W9D1 | Focus: SLAP Sections B, C, D, E  Activity: Trust Fall (Team Building) | Camp Staff | -(B) School Research→ provided an additional handout of local and statewide school statistics to complement participants’ research.  -(C) Identifying School/Community Needs→ simplified resource-mapping handout (e.g., what resources are currently available and what programs/resources are needed).  -(D) Linking School/Community Needs to Action→ Reflected on 2-3 questions only given time.  -(E) S-L project description → Identified a need and a service applicable within their immediate community (e.g., their church or their school); could partner with an existing project or program or develop a new one. |
| W9D2 | Focus: Goal-Setting/ Assessment/ Budget (SLAP Sections F, G, I)  Activity: Human Knot (Team Building) | Camp Staff | -(F) Goal-setting→ Brainstormed project ideas in small groups, present ideas to larger group for feedback, collaboratively decide on an idea, and return to smaller group for planning.  -(G) Assessment→ -Edited handouts (e.g., format, font, visuals, less dense) tailored towards age/grade level to assess the progress and success of the service-learning project.  -(I) Project timeline→ Edited handout (e.g., spacing, font, visuals, less dense, tailored towards age/grade level) to easily map out resources & budget needed for the project. |
| W10D1 | Focus: Planning Final Presentation and Project  Activity: Relay Race (Team Building) | Camp Staff | -Utilized handouts to review various components of the project. |
| W10D2 | Focus: Planning Project Presentation & Celebration Party; Creating Board  Activity: Blind Square (Team Building) | Camp Staff | NA |

*Note*. SLAP=Service-Learning Action Plan; S-L=Service-Learning.

Appendix Figure 1. Reach for the SCI

**REACH FOR THE SCI**

***I am confident, I am courageous, I am choosing a peaceful solution***

Appendix Table 2. Type III Effects from Mixed-Effects Models

|  | Age | | Gender | | Time | |
| --- | --- | --- | --- | --- | --- | --- |
| Outcome | F | p-value | F | p-value | F | p-value |
| ***Youth self-report*** | | | | | | |
| Community belongingness | 0.07 | .80 | 0.59 | .45 | 0.17 | .85 |
| Social responsibility | 5.64 | .03 | 5.27 | .04 | 1.28 | .30 |
| Emotional symptoms | 1.25 | .28 | 9.08 | .007 | 13.06 | < .001 |
| Conduct problems | 0.15 | .70 | 0.12 | .74 | 0.98 | .39 |
| Peer problems | 11.74 | .004 | 1.73 | .21 | 5.96 | .01 |
| Prosocial behaviors | 0.19 | .67 | 2.71 | .12 | 11.00 | < .001 |
| ***Parent report*** | | | | | | |
| Social Skills | 0.75 | .40 | 1.78 | .20 | 2.91 | .09 |
| Externalizing | 0.37 | .56 | 1.09 | .31 | 1.06 | .37 |
| Internalizing | 0.00 | .99 | 1.41 | .25 | 0.47 | .63 |
| ***Staff report*** | | | | | | |
| Social Skills | 1.32 | .27 | 5.01 | .04 | 2.35 | .14 |
| Externalizing | 2.62 | .12 | 5.12 | .04 | 0.06 | .82 |
| Internalizing | 0.15 | .63 | 1.70 | .21 | 11.47 | .003 |
